# Optical spectral transmission to assess glucocorticoid therapy response in patients with arthritis: a longitudinal follow-up comparison with joint ultrasound

**DOI:** 10.1186/s13075-023-03023-9

**Published:** 2023-03-25

**Authors:** Konstantinos Triantafyllias, Tatjana Marinoska, Caroline Heller, Michele de Blasi, Muthuraman Muthuraman, Andreas Schwarting

**Affiliations:** 1 Rheumatology Center Rhineland-Palatinate, Kaiser-Wilhelm-Str. 9-11, 55543 Bad Kreuznach, Germany; 2 Department of Internal Medicine I, Division of Rheumatology and Clinical Immunology, University Medical Center of the Johannes Gutenberg University Mainz, Mainz, Germany; 3 Department of Biomedical Statistics and Multimodal Signal Processing, Neurology, University Medical Center of the Johannes Gutenberg University Mainz, Mainz, Germany; 4 Department of Neurology, University Hospital Würzburg, Würzburg, Germany

**Keywords:** Optical spectral transmission, HandScan, Joint ultrasound, Inflammation, Arthritis follow-up

## Abstract

**Background:**

Optical spectral transmission (OST) is a modern diagnostic modality, able to assess the blood-specific absorption of light transmitted through a tissue, promising quantification of inflammation in the finger and wrist joints of patients with arthritis. To date, there are no adequate data regarding the diagnostic value of OST in the evaluation of inflammatory activity changes, during arthritis follow-up.

Objectives of this study were therefore to examine the performance of OST in assessing response to anti-inflammatory therapy in patients with active arthritis and to explore OST associations with clinical, laboratory, and ultrasonographic (US) activity markers.

**Methods:**

*1173* joints of *54* patients with arthritides of the wrist and finger joints were examined by OST before and after oral administration of glucocorticoids (GC), during a disease flare. For the same time-points patients underwent clinical, laboratory, and joint US [grayscale (GSUS), power-Doppler (PDUS)] examinations. The distribution of ΔOST-values between the two time-points was compared with the respective distributions of ΔPDUS and ΔGSUS by Bayesian statistical analyses. Moreover, the diagnostic performance of OST compared to a control group (*2508* joints of *114* subjects) was examined by receiver operating characteristics and associations of OST values with clinical, laboratory, and arthrosonographic parameters were evaluated by correlation analyses.

**Results:**

OST and US performed similarly in the assessment of inflammatory changes caused by GC (same value-change tendency in *83.2%* of the cases). Bayesian statistics revealed no significant differences between ΔOST and ΔPDUS for all 3 examined joint categories (accuracy: metacarpophalangeal (MCP): *68.1%*; proximal interphalangeal (PIP): *60.4%*; wrists: *50.4%*) and between ΔOST and ΔGSUS for MCP and PIP joints (accuracy: *51.1%* and *78.7%*, respectively). OST diagnostic performance (patients vs. controls) was excellent in both time-points [area under the curve (AUC) before GC=*0.883(95%CI=0.83–0.94)* and after GC=*0.811(95%CI=0.74–0.881); p<0.001*]. Furthermore, OST correlated significantly with all examined sonographic activity scores (all; *p<0.001*) and with swollen joint counts (*p<0.01*).

**Conclusions:**

OST was able to assess response to therapy in a similar way to joint US and correlated significantly with arthritis activity markers. Therefore, OST has proved to be a valuable tool to assist disease activity monitoring in the examined cohort.

**Trial registration:**

German Registry of Clinical Trials, DRKS00016752

## Background

Accurate monitoring of disease activity in patients with arthritides is one of the most important conditions for adequate inflammation control, preservation of joint function, and ultimately outcome improvement [1]. Various independent studies have shown that quantitative rather than subjective monitoring of disease activity can lead to treatment decisions with improved outcomes in patients with arthritis [2]. Furthermore, tight clinical control has been shown to correlate with more effective and longer inflammatory activity suppression in RA and other arthritides, such as psoriatic arthritis (PsA) [3, 4].

In the last years, there has been a longstanding discussion regarding the appropriateness of various diagnostic tools in the assessment of inflammatory activity in patients with different kinds of arthritides [5–8]. Clinical scores such as the “Disease Activity Score 28” (DAS28) can be easily performed and are of undisputable value in the follow-up of RA patients [9]. However, DAS28 is only validated in this condition and can be characterized by a series of further limitations, such as examiner dependence and inadequate estimation of subclinical disease activity. On the other hand, complete clinical assessment by joint ultrasound (US) requires examination of multiple joints, which can be time-consuming, especially in the case of thorough scoring of the US findings [10]. Moreover, there is a need for training and expertise for US examiners [11]. Magnetic resonance imaging (MRI) is not available in every clinical setting, and even if so, high examination costs prohibit frequent follow-up examinations [12, 13]. The use of contrast agents and the usual unilateral performance (i.e., in the case of hand examination) constitute further limitations of this method [13].

Interestingly, a few new arthritis diagnostic methods such as fluorescence optical imaging (FOI) and optical spectral transmission (OST) have been lately introduced in the field of rheumatology. Scarce first data on both of these methods have shown “moderate to good” diagnostic performances regarding the detection of joint inflammation in patients with RA (FOI, OST), PsA (FOI), and JIA (FOI) [14–25]. OST, in particular, promises assessment of joint inflammation by the use of red and near-infrared light technology and thus without the use of a contrast agent or radiation [26]. Moreover, OST examinations can be performed by trained medical assistants and nursing personnel contributing to an easing of the rheumatologic everyday practice. Finally, OST results are quantified automatically by the OST software making the image interpretation process operator independent. Nevertheless, adequate data on this new promising diagnostic modality are missing. Our research group, and others, have examined the diagnostic value of OST in comparison to joint US and have assessed its associations with clinical and laboratory RA activity markers [16, 17, 19, 20]. Moreover, we have evaluated associations of OST not only with disease-associated parameters, but also with patient characteristics and have examined the effect of various possible confounding factors on OST results [16]. In our exploration, we found that OST is associated with clinical, US, and laboratory disease activity markers in a significant manner [16]. Moreover, we showed that patients with RA had higher OST values in comparison to controls even after adjustment for possible influencing factors such as gender and age [16]. However, all OST studies until now have compared this new modality with disease activity markers in only one time-point in the course of the disease. There are no longitudinal data available regarding the ability of OST to detect changes in joint inflammatory activity after the induction of anti-inflammatory medication. Such an exploration would be thought of high importance in order to find out whether this modality can also serve as a valid follow-up tool.

The primary objective of this study was, therefore, to examine the ability of OST to detect response to anti-inflammatory therapy in patients with active inflammatory arthritides of the wrist and/or finger joints. Secondarily, we sought to examine associations of OST with clinical, laboratory, and US disease activity markers, both before and after treatment initiation.

## Patients and methods

### Study population

Fifty-four of 57 recruited consecutive patients with active inflammatory arthropathies were enrolled in the study and were examined by OST during their stay in our inpatient Rheumatology clinic, before and after administration of glucocorticoids (GC). The same patients underwent bilateral US examinations of the MCP, PIP, and wrist joints at the same points in time. We included patients with RA, PSA, peripheral spondyloarthritides, and gout, according to the established classification criteria for every disease [27–30]. Patients had to show clinical signs of synovitis at ≥ 1 joint of both hands, among MCP, PIP, and wrists. Moreover, 114 individuals without underlying inflammatory diseases, arthralgias, or clinical signs of osteoarthritis were recruited consecutively and served as control subjects.

Exclusion criteria in both groups were age<18 years, joint prostheses/implants, severe hand deformities, pronounced ulnar deviation, psoriatic skin plaques on the hands, radiologic or US signs of tophi, recent trauma or surgery, and known photosensitivity. Patients and controls gave their informed consent, and the assessment was reviewed and approved by the local standing committee for ethical conduct, in adherence to the Helsinki Declaration.

### Data collection

We documented epidemiological data (gender, age), anthropometric parameters (weight, height), cigarette smoking, arterial hypertension, and the presence of diabetes mellitus in the patient and the control group. Furthermore, we calculated body mass index (BMI) (kilograms/meters [2]) and the size of both hands (% mean surface covered by two hands divided by % mean surface of the two glass hand rests) in both groups. Counts of tender (TJC) and swollen joints (SJC) before and after GC therapy were assessed by the same trained examiner. Moreover, patient disease activity scores were documented on a visual analog scale (VAS) and erythrocyte sedimentation rate (ESR) was routinely tested and used for the calculation of DAS28-ESR values on both time-points.

### OST measurements

Measurements of OST were performed using the HandScan diagnostic device (Demcon/Hemics®, The Netherlands) by a study nurse, trained in the context of a 2-day education course regarding the use of this modality. The course was held by a certified instructor of Demcon/Hemics® and included a theoretical part and also hands-on training modalities. The OST examiner was blinded with respect to clinical examination, laboratory, and US results, and OST-score calculation was done automatically by the HandScan device.

At the beginning of OST examinations, patient and control subjects placed their forearms on a glass handrest into the HandScan device through two frontal openings that held pressure cuffs. Red and near-infrared laser light at wavelengths of 660nm and 808 nm illuminated the palmar side of the distal forearm [both wrists, MCP, PIP, and reference areas for every joint]. Light transmitted through the hands was recorded by a camera placed at the upper side of the device.

A complete measurement lasted approximately 100 s and consisted of 3 phases: (a) a low cuff pressure phase, (b) an increased cuff pressure phase [55 mmHg (=7.3 kPa)], and (c) a low cuff pressure phase. During the first phase, baseline transmission was assessed. In the second phase, increased cuff pressure caused blood to pool in the examined areas. During the third phase, inversion of venous occlusion and blood pooling took place.

A built-in software allowed the automatic identification of “regions of interest” (ROI: wrists, MCP I-V, and PIP I-V in both sides) and reference areas, which were located distally to the examined joints. A comparison between the blood flow in the ROI and in the reference areas served as a control mechanism for the presence of impaired or increased peripheral blood flow due to systemic factors, such as body temperature, diabetes mellitus, nicotine use, or vasoactive medication.

As described previously, OST assessed joint hypervascularity in accordance with known semiquantitative power Doppler US (PDUS) scoring methods [31] and translated it to a grade between 0 and 3 (“0” meaning none hypervascularity and “3” standing for the highest possible grade of hypervascularity). In order to calculate an overall OST score, single-joint OST scores were added and multiplied by the number of joints measured. Normally, this number is “22” (10 MCP, 10 PIP, and 2 wrists), but it can also be lower in cases of pre-existing finger amputation or anatomic joint anomalies that make identification of the joint from the OST software impossible. Finally, the software divided this count through 22 to get a weighted average score of both hands, thus resulting in values between 0 and 66. OST data translation was automatically performed by the HandScan device.

Intra-observer variability of OST was tested in patients of our study group by intra-class correlation (ICC) analyses both via randomly selected and averages of independent measurements and showed excellent correlations (random: ICC = 0.82, 95% CI: 0.61 to 0.93, *p*=0.0029; averaged: ICC = 0.98, 95% CI: 0.76 to 0.96, *p*=0.029).

### Ultrasound examination

US protocol was in accordance with the EULAR guidelines regarding the positioning of the patient and scanning planes [32]. A linear transducer (4–13 MHz) of a MyLab70 US device (Esaote, Italy) operating at the maximal frequency of 13 MHz was used for the measurements. Both grayscale US (GSUS) and PDUS examinations were performed at the dorsal aspects of MCP I-V, PIP I-V, and the wrists (radiocarpal/midcarpal joint recesses) of every patient [16, 20].

Color gain was set at the disappearance of color noise and the pulse repetition frequency (PRF) was set as low as possible to have maximum sensitivity resulting in a frequency of about 750 Hz. PDUS semi-quantitative scoring was based on the methodology suggested by Szkudlarek et al. and later grossly endorsed by EULAR-OMERACT [31, 33]. Thus, PDUS was graded semiquantitatively as follows: (A) grade 0 = no flow in the synovium; (B) grade 1 = single vessel signals; (C) grade 2 = confluent vessel signals in less than half of the area of the synovium; (D) grade 3 = vessel signals in more than half of the area of the synovium.

Presence of synovitis/joint effusion on GSUS was scored using a binary scoring method [34, 35]. Finally, tenosynovitis of the wrist flexors and extensors was examined in B-mode-US and was defined as an abnormal anechoic or hypoechoic widening of the tendon sheath [36]. Its presence or absence was documented in a binary manner [19]. All US examinations were performed by a blinded experienced examiner [K.T., Rheumatologist, certified US trainer of the German Society of Ultrasound in Medicine (D.E.G.U.M.)].

### Statistical analysis

The assumption of normality of distribution was evaluated by the Shapiro-Wilk test and a graphic method (quantile-quantile plots). Comparison of categorical variables was performed by a chi-squared test. Differences between patient and control groups were evaluated by Mann-Whitney *U* (skewed variable distributions) or *t*-test (normal variable distributions). These tests were also applied to analyze associations between OST and binary categorical variables in both groups. Moreover, Spearman’s and Pearson’s correlation coefficients rho and *r* were used to assess correlations of OST and continuous characteristics in the 2 groups. Additionally, receiver operating characteristics (ROC) were performed in order to assess the ability of OST to differentiate between control subjects and patients.

Finally, a comparison of distributions of inflammatory activity changes assessed by the two examination methods (ΔOST vs. ΔPDUS and ΔOST vs. ΔGSUS) was performed by Bayesian posterior analysis using the Markov Chain Monte Carlo approach for the choice of priors; the default Markov Chain Monte Carlo sample size of 100,000 was used for all analyses. Group differences were calculated as differences in the means and are shown as differences between the groups, which describes the ability to separate the compared groups. Differences with an accuracy ≥80% were interpreted as significant (*p<0.05). Accuracies ≥90% were regarded as highly significant (**p<0.01), while differences above 95% between the group values were assigned the highest significance (***p<0.001). An 80% accuracy for differentiating between two groups is defined by a 20% probability of rejecting a true null hypothesis. This corresponds to a *P* value of *p*=0.05 [37], hence our cut-off. The BEST R package (https://jkkweb.sitehost.iu.edu/BEST/) was used for the estimation of the Bayesian posterior distributions. All other statistical calculations were performed using the SPSS software 23.0.

## Results

OST measurements were performed in a total of *1173* joints of *54* patients with active inflammatory arthritides [*39* with RA, *11* with peripheral seronegative arthritides (PsA/peripheral SpA), and *4* with gout] and in *2508* joints of *114* control subjects (female patients: *66.7%* vs. female controls: *77.2%*, *p>0.05)*. *Fifteen* joints of the patient group were automatically excluded by the OST software, due to anatomic anomalies/missing fingers. US examinations of the MCP, PIP, and wrist joints were performed in the patient group in a total of *1188* joints.

Oral glucocorticoids were administered in almost all of the cases at a starting dosage of *0.5* mg/kg. In only a few exceptions (approximately 5% of the cases), a higher dosage (~ 1mg/kg) was selected due to high disease activity (DAS28>5.1). The cumulative doses of prednisolone pulse therapy had a median of *115* mg (*80–150*, IQR) and the mean duration of pulse therapies, as well as the interval between the two examination time-points, were *4* (*3–5*, IQR) days. OST and US were performed on the same day for both examination time-points. Median DAS28 was *5.12 (4.33–6.10)* previous to GC and *3.85 (3.40–4.82)* after GC and median disease duration was *3 (0.5–3,* IQR*)* years.

Descriptive characteristics of both groups are presented in Tables 1 and 2.

**Table 1:** Descriptive characteristics of patients for both examination time-points

Patient characteristics	***Time-point****(a)*	***Time-point****(b)*	***Significance****(p)*
Patient count (*n*)	54	54	-
Age (years)^b^	62.91 ± 12.68	-	-
Disease duration (years)^†^	3 (0.5–3.0)	-	-
BMI (kg/m^2^)^b^	28.40 ± 4.70	-	-
Gender (female), %	66.7%	-	-
Handsize, %	67.71 ± 7.94	-	-
ESR, mm/h^a^	29.0 (17.25–50.00)	20.00 (13.50–34.25)	0.001**
DAS28^a^	5.12 (4.33–6.10)	3.85 (3.40–4.82)	<0.001***
PDUS^†a^	8.75 (5.38–16.25)	4.75 (2.38–8.63)	<0.001***
GSUS^a^	6.00 (3.00–8.00)	3 (2.00–3.00)	<0.001***
OST^b^	18.33 ± 4.69	16.43 ± 4.86	<0.001***
TJC^a^	6.00 (2.00–12.00)	2.00 (0.00–5.25)	<0.001***
SJC^a^	6.00 (3.00–9.00)	4.00 (2.00–6.00)	<0.001***
VAS^a^	67.5 (48.75–76.25)	40.00 (30.00–51.25)	<0.001***
Tenosynovitis, %	66.7%	33.3%	<0.001***
Arterial hypertension (yes), %	48.1%	-	-
Diabetes mellitus II (yes), %	9.3%	-	-
Nicotine (yes), %	24.1%	-	-

^a^Data are presented as median (interquartile range) as not normally distributed

^b^Data are presented as mean (± SD) as normally distributed

*t*-test (normal distribution) and Mann-Whitney *U* test (skewed distribution) were used to investigate the relationships between OST and qualitative patient characteristics

*BMI *Body mass index, *ESR *Erythrocyte sedimentation rate, *DAS28 D*isease activity score 28, *PDUS *Power Doppler ultrasound score, *GSUS G*rayscale ultrasound score, *OST *Optical spectral transmission, *RF *Rheumatoid factor, *TJC *Tender joint count, *SJC S*wollen joint count, *VAS *Visual analog scale

**p < 0.01, ***p < 0.001

**Table 2:** Descriptive characteristics of patients vs. controls

	***Controls****(n=114)*	***Patients****(n=54)*	***Significance****(p)*
Age (years)^†^	51 (35–57)	61 (56–70.25)	<0.001***
BMI (kg/m^2^)^a^	25.50 (21.72–28.55)	28.38 (25.33–31.21)	0.001***
Gender (female), %	77.2%	66.7%	0.104
OST (*time-point a*)^b^	10.79 ± 4.20	18.33 ± 4.69	<0.001***
OST (*time-point b*)^b^	10.79 ± 4.20	16.43 ± 4.86	<0.001***
Arterial hypertension, %	19.4%	48.1%	<0.001***
Diabetes, %	1.9%	9.3%	0.048*
Nicotine, %	19.4%	24.1%	0.314

^a^Data are presented as median (interquartile range) as not normally distributed

^b^Data are presented as mean (± SD) as normally distributed

*t*-test (normal distribution) and Mann-Whitney *U* test (skewed distribution) were used to investigate the relationships between OST and qualitative patient characteristics

*BMI *Body mass index, *OST *Optical spectral transmission

*p < 0.05, **p < 0.01, ***p < 0.001

Mean OST values were significantly higher in the patient group for both time-points, compared to the control group [controls: *10.79 ± 4.20* vs. patients at time-point a: *18.33 ± 4.69* and patients at time-point b: *16.43 ± 4.86*; both *p<0.001*] (Table 2).

### Comparison of diagnostic performance during follow-up between US and OST

OST and PDUS performed similarly in the assessment of inflammatory changes caused by GC, given the fact that the same value-change tendency (i.e., decrease in OST with parallel decrease in PDUS after GC pulse therapy) could be observed in *83.2%* of the cases.

**Fig. 1:**
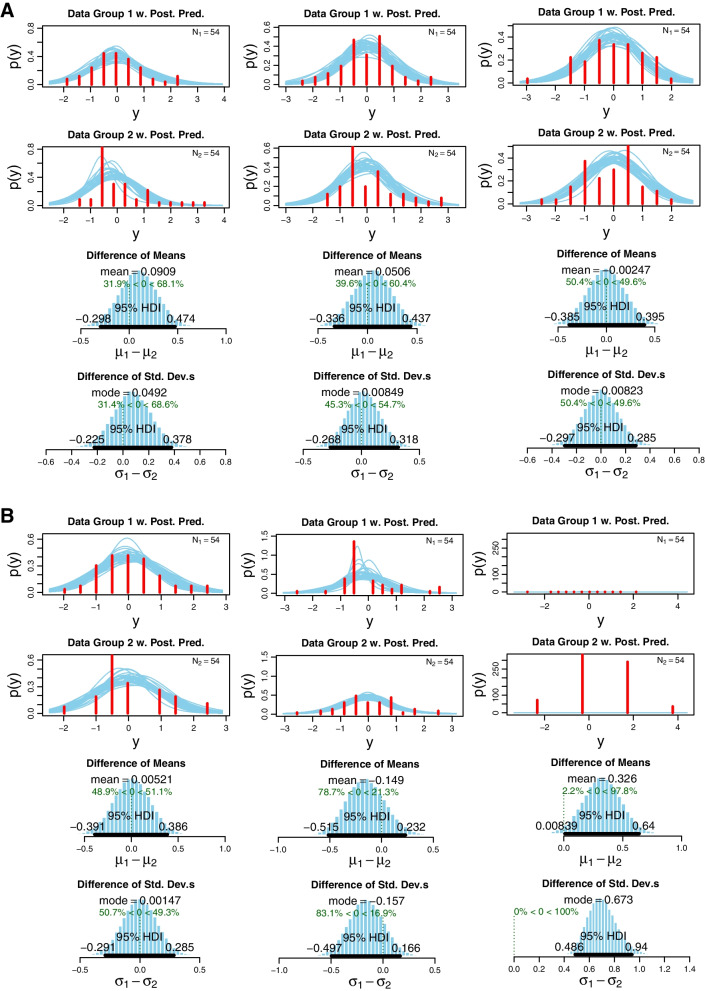
**A**, **B** Bayesian statistic analysis: **A** ΔPDUS vs. ΔOST (left) and **B** ΔGSUS vs. ΔOST (right). Bayesian statistics of the posterior distribution (PD) for the variables PDUS (**A**) and GSUS (**B**), respectively. The first columns of 1A and 1B (top to bottom) show the group 1 raw values distribution and group 2 raw values distribution followed by the difference of the means and standard deviation. For all the PD graphs, the 95% highest density interval is shown as dark black lines. The first column represents the MCP, the second column PIP, and the third column wrists respectively. Bayesian analysis, accuracy was defined as *(*p* < 0.05) >80%, **(*p* < 0.01)>90%, ***(*p* < 0.001)>95%. *PDUS*, power Doppler ultrasound; *GSUS*, grayscale ultrasound; OST, optical spectral transmission

Furthermore, Bayesian statistics revealed no significant differences between the distributions of ΔOST and ΔPDUS for all 3 examined joint categories (accuracy: MCP: *68.1%*; PIP: *60.4%*; wrists: *50.4%*) (Fig. 1A). In the case of comparisons between the distributions of ΔOST and ΔGSUS values, no statistical differences regarding MCP and PIP (accuracies: *51.1%* and *78.7%* respectively) joints could be observed (Fig. 1B). On the contrary, distributions of ΔOST and ΔGSUS values were statistically different in the case of the wrist joints (97.8%***), thus pointing to a possible different performance of the two methods in detecting GSUS changes in larger joints (Fig. 1B). This could be also implicated by the fact that the tendency of GSUS and OST value changes was the same in roughly two-thirds of the cases (approx. 65%).

### Receiver operating characteristics

**Fig. 2:**
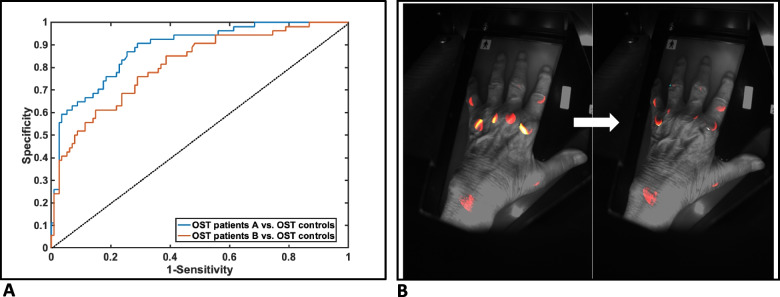
**A**, **B** Diagnostic performance of optical spectral transmission. **A** Receiver operating characteristics: OST patients vs. OST controls in time-points A and B. (i) Area under the curve: 0.883 (95%CI 0.82–0.94); sensitivity 0.91, specificity 0.71; ***p < 0.001. (ii) Area under the curve: 0.81 (95%CI 0.74–0.881); sensitivity 0.76, specificity of 0.71; ***p < 0.001. **B** OST examination results before (**A**) and after (**B**) glucocorticoid pulse therapy in a patient with rheumatoid arthritis during a disease flare (OST score A=18.1; OST score B=14.3). OST, optical spectral transmission

ROC were performed in order to evaluate the comparison “patients vs. controls” via OST, both before and after the initiation of GC pulse therapy. Diagnostic performance of OST was excellent in both time-points. The area under the curve (AUC) before GC pulse therapy was *0.883 (95%CI 0.82–0.94)*, with a sensitivity of *0.91* and a specificity of *0.71*, for an OST cut-off of *12.74* (Youden index *0.618*) (Fig. 2). After GC pulse therapy AUC was *0.81 (95%CI 0.74–0.881)*, with a sensitivity of *0.76* and a specificity of *0.71*, for an OST cut-off of *12.69* (Youden index *0.47*) (Fig. 2).

### Correlations of OST with sonographic and clinical activity markers

**Fig. 3:**
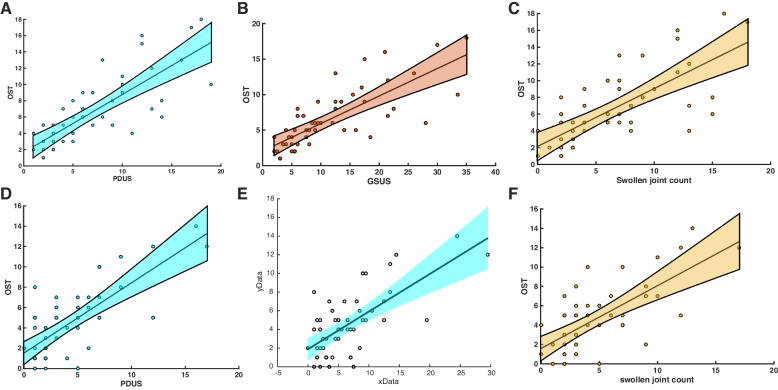
Associations of OST with PDUS, GSUS, and swollen joint counts for both examination time-points [**A**, **B**, **C**: time-point (a) and **D**, **E**, **F**: time-point (b)]. All, *p<0.05. OST, optical spectral transmission; PDUS, power-Doppler ultrasound; GSUS, grayscale ultrasound

OST correlated moderately/strongly with PDUS and GSUS at both examination time-points: PDUS (a) *rho=0.449* and (b) *rho=0.414*, both; *p<0.001* and GSUS (a) *rho=0.494*, *p=0.0002* and (b) *rho=0.56*, *p<0.0001* (Fig. 3, Table 3).

**Table 3:** Associations of OST with patient- and disease-associated characteristics

	**Time-point (a)**		**Time-point (b)**	
	*Rho*	*Significance p*	*Rho*	*Significance p*
Age (years)^b^	0.043	0.760	-	-
BMI^b^	*0.315*	0.021*	-	-
Handsize, %^b^	0.136	0.331	0.201	0.150
ESR, mm/h^a^	0.041	0.768	0.057	0.683
DAS28^a^	0.239	0.081	0.160	0.248
PDUS^a^	*0.449*	0.001**	*0.414*	0.002**
GSUS^a^	*0.494*	0.0002***	*0.56*	<0.0001***
Tenosynovitis^a^	*−0.059*	*0.688*	*−0.025*	*0.857*
TJC^a^	*−*0.064	0.645	*−*0.077	0.581
SJC^a^	*0.379*	0.005**	*0.382*	0.004**
VAS^a^	0.135	0.330	-0.016	0.910
	**Tme-point (a)**		**Time-point (b)**	
	*Median* ± SD	*Significance p*	*Median* ± SD	*Significance p*
Gender^b^				0.320
*Female*	18.15 ± 4.80	0.687	15.96 ± 4.86	
*Male*	18.69 ± 4.57		17.38 ± 4.85	
Arterial hypertension^b^				0.849
*No*	18.91 ± 4.23	0.359	16.56 ± 4.66	
*Yes*	17.72 ± 5.15		16.30 ± 5.15	
Diabetes mellitus II^b^				0.844
*No*	18.46 ± 4.82	0.369	16.41 ± 5.07	
*Yes*	16.98 ± 3.06		16.63 ± 1.80	
Nicotine^b^				0.412
*No*	17.81 ± 4.24	0.230	16.10 ± 4.68	
*Yes*	16.09 ± 17.50		17.50 ± 5.43	

Spearman’s (^a^not normal distribution) and Pearson’s (^b^normal distribution) tests were performed to investigate the relationships between OST and quantitative patient characteristics. *t*-test was used to investigate the relationships between OST and qualitative patient characteristics. ^b^Data are presented as mean (± SD), as normally distributed

*BMI* Body mass index, *ESR* Erythrocyte sedimentation rate, *DAS28* Disease activity score 28, *PDUS* Power Doppler ultrasound score, *GSUS* Grayscale ultrasound score, *TJC* Tender joint count, *SJC* Swollen joint count, *VAS* Visual analogue scale

*p < 0.05, **p < 0.01, ***p < 0.001

Similarly, OST correlated moderately with swollen joint counts at both time-points [(a) *rho=0.417*, *p=0.002* and (b) *rho=0.382*, *p=0.004*, respectively] (Fig. 3, Table 3). DAS28 showed solely a trend of association with OST (*rho=0.24*, *p=0.08*) at the examination time-point (a) (before GC administration). The relationships between OST and tender joint count or ESR did not reach the appropriate level of statistical significance.

Moreover, OST correlated among patients moderately with BMI (*rho=0.315*, *p=0.021*). There were no statistically significant relationships between OST and nicotine use, arterial hypertension, diabetes mellitus, and gender (all, *p>0.05*; Table 3).

Regarding the control group, OST correlated moderately/strongly with hand size (*rho=0.477*, *p<0.001*) and poorly with BMI (*rho=0.24*, *p=0.015*). Furthermore, male controls showed higher OST values than females (*p<0.001*).

## Discussion

Our data suggest that OST is a promising diagnostic tool with a possible utility in the follow-up of patients with RA. Moreover, we found an excellent diagnostic performance in the comparison between controls and patients and moderate-strong significant correlations between OST and several clinical and joint US activity markers.

To our knowledge, this is the first study to examine the diagnostic value of OST in the follow-up of patients with active arthritis, taking as a reference the gold standard assessment method of joint US, which also allows the detection of subclinical disease activity.

In the past, several studies, including those of our working group have shown that OST could be a promising diagnostic modality for the screening of arthritis patients, particularly when certain limiting parameters, such as OST confounding factors, are taken into account [16, 17, 19–21, 38]. However, longitudinal data regarding the ability of OST to follow up the joint inflammatory changes in patients under anti-inflammatory therapy are scarce. Until now, there has been only one study published on the topic, which however examined correlations of OST with solely clinical parameters (DAS28), during a time period of 6 months [39]. In this study, a longitudinal association of OST scores with DAS28 could be found. Nevertheless, no significant predictive ability of OST regarding treatment response at 3 and 6 months was established. A possible reason could be the fact that response on therapy was defined by an improvement of DAS28, which however includes further joints (knees, elbows, shoulders) and additional items (VAS, inflammation markers), which are not included in OST scoring.

In our study, we have followed a more targeted approach and have used joint-US as a diagnostic reference for OST. This gave us the possibility to assess inflammatory changes by OST of the exact same joints examined by US and thus obtain a more tailored evaluation of OST. Moreover, joint US gave us the opportunity to assess subclinical disease activity, which can predict relapse after premature withdrawal of anti-inflammatory therapy and is thus of higher diagnostic value compared to clinical examination [40, 41]. Furthermore, we have applied an advanced statistical methodology (Bayesian statistics) which allowed us to compare distributions of the ΔPDUS and ΔGSUS with the respective distributions of ΔOST during follow-up in a thorough manner. Through this methodological approach, we could find that distributions of ΔPDUS and ΔOST data were not statistically significantly different during follow-up for all 3 examined joint categories (wrist, MCP, PIP). Thus, a similar diagnostic potential for OST and PDUS can be postulated.

This finding is important, given the fact that valid tools of arthritis follow-up assessments are greatly needed in the field of Rheumatology. As well-known, a tight clinical control strategy associates with a longer activity suppression and an improved overall prognosis [42]. Of course, the high utility of US in everyday practice remains undisputable. US should be always considered both for the diagnosis and the follow-up of inflammatory arthropathies and can guide therapeutic decisions by assisting the monitoring of inflammatory activity [43, 44]. Moreover, US allows assessment of other anatomic districts except for hands and wrists, as well as evaluation of topographic inflammation distribution at tissue level [13].

On the other hand, US can be time-costly, is often performed only by physicians, and is additionally examiner-dependent. By having a tool with similar diagnostic value to US without the aforementioned disadvantages, everyday rheumatology practice could be eased.

Interestingly enough, the distribution of ΔOST was similar to the distribution of ΔGSUS of only the MCP and PIP joints and not the wrists*.* This finding can point out to a possible reduced diagnostic sensitivity of OST in detecting B-mode abnormalities at the wrist joint level and/or be explained by the known tendency of slower GSUS changes compared to PDUS [45]. Interestingly, diagnostic performance of OST at the wrist level has been previously described to be worse compared to MCP and PIP joints [20]. More robust bony and joint structures of the wrist, compared to small joints, could provide a possible explanation for this result [16, 20]. However, an improvement of diagnostic performance at the wrist level was reported after the installation of a new light source [19], and in our study, we have used the most modern HandScan version. As a matter of fact, in both the present and a previous OST study of our group, a better overall diagnostic performance at the wrist joint level compared to small joints could be found [16]. However, this comparison included a definition of joint inflammation based on a combination of both PDUS and GSUS values and thus cannot be used to draw conclusions regarding sole B-mode US changes. In general, OST seems to have an acceptable diagnostic performance during follow-up and associated stronger with PDUS changes than with GSUS changes caused by synovial thickening and/or joint effusion. Based on the background technologies of power Doppler and OST, which focus on the detection of vascularity changes, this result seems plausible.

ROC analyses showed an excellent performance of OST in distinguishing arthritis patients from controls at both examination time-points. In fact, AUC, sensitivity, and specificity values were higher compared to our previous study [16]. The reason for that could be the fact that in the present exploration patients with a higher mean disease activity were included. Interestingly, the established associations of OST with both the GSUS and the PDUS scores were also similar to our previous work [16]. Further working groups came to the same conclusion [20], suggesting that not only PDUS, but also GSUS should be taken into consideration when comparing OST with US. As would be expected, OST correlated significantly with the count of swollen joints and not with the count of tender joint counts or VAS, pointing to the fact that OST detects inflammation and not its “byproducts”.

This study has some limitations. OST measures inflammation only in the wrist, MCP, and PIP joints. Thus, the inflammatory activity of the DIP could not be assessed by OST. For that reason, we have not included any US values of the DIP joints in our assessed PDUS and GSUS scores. Despite this limitation, we could show positive proof of principle regarding the validity of follow-up assessments. Furthermore, even though the presence of tenosynovitis was examined via GSUS in all patients, we have decided to focus on synovial inflammation and compare joint OST scores with their respective US joint scores. The reason for that was the fact that OST is not validated in the assessment of tenosynovitis and that no tendon-specific scores are provided by the HandScan device. Interestingly, no association between tenosynovitis and OST was found in this cohort. Nevertheless, further examinations of the role of tendon inflammation are needed also in order to rule out an interference of tendon inflammation with OST assessment.

Another possible limitation arises from the fact that a 4–13-MHz US probe was used and this could have led to reduced sensitivity, in particular in the area of small joints. However, our probe was operating at a frequency of 13 MHz and is thus comparable with the one used by the EULAR-OMERACT task force [33]. Moreover, the objective of the study required the inclusion of patients with high disease activity and that could have led to over-estimation of diagnostic accuracy. Interestingly, OST has also been examined in cohorts of RA patients with lower disease activity and has shown acceptable diagnostic performances, which were however lower than this of the present study [16, 17, 19, 20]. Thus, there is a need for further follow-up studies that will evaluate OST performance in patients with also lower disease activity states. Finally, the slight variability among patients in terms of duration/dosage of glucocorticoids and examination time-points should be also taken into account. Nevertheless, OST and US were performed on the same days making valid comparisons of these two methods feasible.

## Conclusions

To summarize, the here-presented data indicate a good diagnostic performance of OST in the follow-up evaluations of patients with inflammatory arthritis. OST was able to assess response to therapy in a similar way to joint US and correlated significantly with arthritis activity markers. Joint US remains of course gold standard in the follow-up examinations of patients with arthritis, given its high sensitivity and overall diagnostic accuracy. However, OST can prove to be a valuable non-invasive, time- and resource-saving tool to also assist the monitoring of disease activity. Further data on this modern diagnostic modality are thus greatly needed.

## Data Availability

The data analyzed during the current study are available from the corresponding author on reasonable request.

## Abbreviations

OST: Optical spectral transmission
US: Ultrasound
GC: Glucocorticoids
GSUS: Gray-scale ultrasound
PDUS: Power Doppler ultrasound
AUC: Area under the curve
RA: Rheumatoid arthritis
PsA: Psoriatic arthritis
DAS28: Disease Activity Score calculated on 28 joints
MRI: Magnetic resonance imaging
FOI: Fluorescence optical imaging
MCP: Metacarpophalangeal joints
PIP: Proximal interphalangeal joints
BMI: Body mass index
TJC: Tender joint count
SJC: Swollen joint count
ACR: American College of Rheumatology
EULAR: European League Against Rheumatism
VAS: Visual analog scale
ESR: Erythrocyte sedimentation rate
ROI: Regions of interest
ROC: Receiver operating characteristic
